# Cardiac Alarmins as Residual Risk Markers of Atherosclerosis under Hypolipidemic Therapy

**DOI:** 10.3390/ijms231911174

**Published:** 2022-09-22

**Authors:** Viorel I. Suica, Elena Uyy, Luminita Ivan, Raluca M. Boteanu, Aurel Cerveanu-Hogas, Rune Hansen, Felicia Antohe

**Affiliations:** 1 Institute of Cellular Biology and Pathology “Nicolae Simionescu”, 050568 Bucharest, Romania; 2 SINTEF Digital, 7465 Trondheim, Norway; 3 Department of Circulation and Medical Imaging, Norwegian University of Science and Technology, 7491 Trondheim, Norway

**Keywords:** atherosclerosis, alarmins, residual risk, proteomics, mass spectrometry

## Abstract

Increased levels of low-density lipoproteins are the main risk factor in the initiation and progression of atherosclerosis. Although statin treatment can effectively lower these levels, there is still a residual risk of cardiovascular events. We hypothesize that a specific panel of stress-sensing molecules (alarmins) could indicate the persistence of silent atherosclerosis residual risk. New Zealand White rabbits were divided into: control group (C), a group that received a high-fat diet for twelve weeks (Au), and a treated hyperlipidemic group with a lipid diet for eight weeks followed by a standard diet and hypolipidemic treatment (atorvastatin and PCSK9 siRNA-inhibitor) for four weeks (Asi). Mass spectrometry experiments of left ventricle lysates were complemented by immunologic and genomic studies to corroborate the data. The hyperlipidemic diet determined a general alarmin up-regulation tendency over the C group. A significant spectral abundance increase was measured for specific heat shock proteins, S100 family members, HMGB1, and Annexin A1. The hypolipidemic treatment demonstrated a reversed regulation trend with non-significant spectral alteration over the C group for some of the identified alarmins. Our study highlights the discriminating potential of alarmins in hyperlipidemia or following hypolipidemic treatment. Data are available via ProteomeXchange with identifier PXD035692.

## 1. Introduction

Atherosclerosis is a multifactorial chronic inflammatory disease of the medium and large caliber blood vessels with fatal repercussions if drug-based therapy and a change in lifestyle are not taken into consideration. A high-fat diet, hypertension, smoking, diabetes, and sedentarism are all risk factors that must be dealt with in due time before a critical threshold in disease progression is crossed. The most common denominator, however, which is referred to in the treatment of cardiovascular diseases, is the elevated levels of low-density lipoprotein cholesterol (LDL-C) [1].

Statins are the most prescribed drugs during lipid-lowering therapy. Although they work effectively in inhibiting the de novo synthesis of cholesterol, there is a residual risk of cardiovascular events in patients prescribed statin treatment [2,3]. A more recent and encouraging strategy, however, in the management of LDL-C levels, has been represented by the proprotein convertase subtilisin/kexin type 9 (PCSK9) inhibition-based therapy [4]. PCSK9 plays a key role in plasma cholesterol metabolism by regulating the low-density lipoprotein receptor’s (LDLR) hepatic level through the activation of its lysosomal degradation route [5]. The direct involvement of PCSK9 in lipid metabolism was originally observed when documenting the gain of function mutation of the PCSK9 gene, lowering the LDLR in the liver, which, in turn, translates to high levels of circulating LDL-C and increased susceptibility to coronary heart disease [6]. The reverse has also been confirmed where the loss of function of PCSK9 mutations would have a beneficiary outcome [7]. A standalone statin therapy is effective up to a point in lowering LDL-C levels, especially due to the paradoxical effect of statin administration in relation to PCSK9. The cause of this is the simultaneous regulation of both PCSK9 and LDLR by cholesterol, which determines that a standalone statin therapy induces up-regulation of PCSK9 [8]. Therefore, an argument in favor of a synergistic statin and PCSK9 inhibition therapy was postulated and readily confirmed in several medical studies [4,9,10]. One such example is represented by the GLAGOV Randomized Clinical Trial from 2016 which outlined the benefits of using a combined statin and PCSK9 inhibitor administration for atherosclerosis regression. The study proved that the therapy not only decreased the level of LDL-C compared to the placebo, but also demonstrated, using intravascular ultrasound technology, that the total atheroma volume decreased [4].

Alarmins or damage-associated molecular patterns (DAMPs) are a class of endogenous proteins without apparent structural similarities, but with a common characteristic of activating the innate immune system [11]. By active secretion or passive release from dying or damaged cells, alarmins bind specific receptors of immune cells initiating and propagating an inflammatory response. They serve, thus, as danger signals, alerting the organism that a trauma or a pathogen infection has occurred. In the case of myocardial ischemia, as a result of coronary occlusion, a plethora of alarmins are locally and systemically released from dying cardiomyocytes, initiating an inflammatory response for early tissue debridement by neutrophils and inflammatory macrophages [12]. Alarmins, such as high mobility group box-1 (HMGB1) or the S100 family of proteins, have been documented as active players in the atheroma micro-environment or in ischemic events [13]. HMGB1, for example, has been investigated in subclinical coronary artery disease (CAD) where a potentiating inflammatory effect was attributed to the alarmin in the acute phase of ischemic injury [14]. The S100 A8/A9 heterodimer alarmin (calprotectin) has recently gained popularity through its demonstrated critical importance in post-myocardial infarction in the early, inflammatory phase [15,16]. Its level rapidly increases in the myocardium and within systemic circulation, binding to specific receptors in order to enhance the recruitment of polymorphonuclear neutrophils and monocytes.

High-resolution mass spectrometry-based proteomics has the unique advantage of both high-throughput and sensitivity, characteristics often regarded as critical in untargeted screening for markers associated with disease progression and treatment efficacy [17]. In the recent decade, numerous proteomic studies have unraveled the differentiated levels of specific alarmins in various inflammatory diseases [18]. However, reports of coherent alarmin patterns in hyperlipidemic conditions are scarce. In our study, we purposely used an advantageous atherosclerotic animal model, a modern hypolipidemic treatment, and high-resolution mass spectrometry to distill alarmins specific for advanced hyperlipidemia and stabilizing treatment in order to reveal whether a residual risk can be associated with specific danger signal patterns. Herein, we report a quantitative tableau of alarmins, which, in the majority of cases, are characterized by significant regulation caused by the hyperlipidemic condition and where the hypolipidemic treatment levels out the regulating effect. We also report a number of alarmins that were not affected by the hypolipidemic treatment and that possess the same regulation tendency as advanced hyperlipidemia, which may be associated with a residual risk.

## 2. Results

### 2.1. Characterization of the Experimental Model

**Figure 1:**
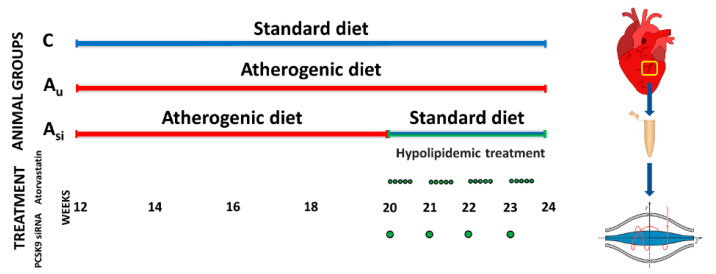
Animal experimental workflow presenting the three groups of New Zealand White rabbits under a standard diet for three months (Control group: C), under a hyperlipidemic diet (standard diet supplemented with 0.5% cholesterol and 5% corn oil) for the same amount of time (atherosclerotic group: Au), and a group of rabbits which, after two months of hyperlipidemic diet, were switched to a standard diet for the last month together with the administration of a daily oral dose of atorvastatin and a weekly IV dose of PCSK9 siRNA (Asi). The animals’ euthanasia was performed after three months of experimentation followed by blood and organ harvesting. Inter-ventricular myocardial tissue, containing part of the left-descending coronary artery, was mechanically homogenized and used for downstream assays, including high-resolution orbitrap-based mass spectrometric proteomic analysis.

Challenged to a high-fat diet, New Zealand White rabbits are a widely used laboratory model for atherosclerosis. The close resemblance of its lipoprotein metabolism with that of humans [19] makes the animal susceptible to developing atherosclerotic plaques in the lesion-prone areas of the vascular bed after prolonged high levels of circulating low-density lipoproteins. In this regard, we designed an experimental workflow where rabbits were either challenged to a hyperlipidemic diet or were administered a synergic hypolipidemic treatment (Figure 1).

**Figure 2:**
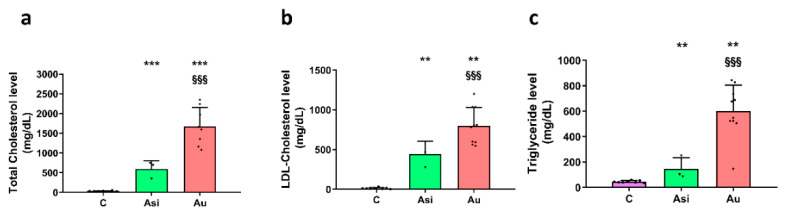
Serological measurements of total cholesterol (**a**) LDL-cholesterol, (**b**) triglycerides, (**c**) from control: C, PCSK9 siRNA and statin-treated hyperlipidemic: Asi and advanced hyperlipidemic: Au samples. The statistical significance over C samples is represented by * (** *p* < 0.01, *** *p* < 0.001), while the one over Asi is visible with § (§§§ *p* < 0.001).

After twelve weeks of dietary fats, the animals from the Au group presented significant alterations in the serum lipid profile: a 48-fold increase in total serum cholesterol, a 62-fold increase in LDL-C, and a 13-fold increase in triglycerides (Figure 2). The combined hypolipidemic treatment, which was applied after two months of a high-fat diet, resulted in increased biochemical sera values (vs C group), but was significantly lower when compared to the Au group: a 2.8-fold and 1.8-fold decrease in total cholesterol and LDL-C, respectively, and a 4.1-fold lower triglycerides level (Figure 2).

**Figure 3:**
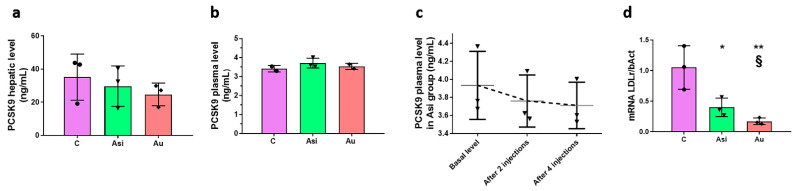
PCSK9 treatment evaluation in the liver (**a**) and plasma (**b**) harvested after animals’ euthanasia. Evolution of PCSK9 level in the plasma of animals receiving statin and PCSK9 siRNA treatment (**c**). The direct target of PCSK9, the hepatic receptor for LDL (LDLr) was also evaluated after a high-fat diet and hypolipidemic treatment (**d**). The statistical significance over C samples is represented by * (* *p* < 0.05, ** *p* < 0.01), while the one over Asi is visible with § (§ *p* < 0.05).

The Asi animals received a combined hypolipidemic treatment for 1 month concomitantly with a standard diet following two months of high-fat intake. We performed specific ELISA assays to determine the PCSK9 absolute level in both the hepatic and plasma samples collected from 3 animals per biological group. Although a small decrease (~15%) in the tissue samples was observed in the Asi group when compared to C, the difference was not statistically significant (Figure 3a). The plasma samples revealed a ~10-fold smaller concentration than the hepatic ones, but, similarly, no significance was discerned between the groups (Figure 3b). When performing a temporal profile of the PCSK9 protein level from the plasma of the Asi animals, a non-significant decrease after 2 and 4 injections, respectively, was revealed when compared to the basal level prior to hypolipidemic treatment administration (Figure 3c). The LDL receptor (LDLr) was also evaluated in the three conditions and we found significantly lower levels in both Asi and Au conditions as opposed to the control one. However, a significantly higher mRNA expression was obtained for the Asi group when compared to the Au group (Figure 3d).

**Figure 4:**
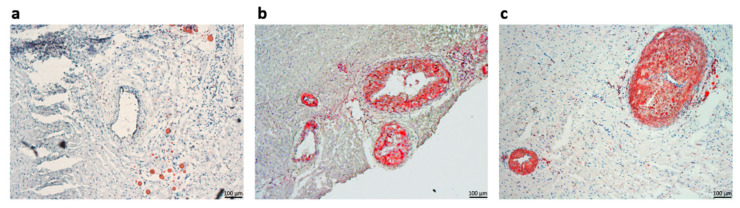
Representative hematoxylin and Oil Red O staining of left-descending coronary artery sections showing nuclei and lipid deposits, respectively, for control-C (**a**), PCSK9 siRNA-treated hyperlipidemic animals-Asi (**b**), and the advanced hyperlipidemic animals-Au (**c**).

Serial sectioning of inter-ventricular myocardial tissue was performed, followed by Oil Red O staining in order to reveal lipid deposits in the left anterior-descending coronary artery. Substantial atherosclerotic deposits were observed in the Asi samples, where a subendothelial accumulation of lipids and intimal thickening were observed (Figure 4b). However, in the case of the Au samples, extensive areas of endothelial-, macrophage-, and possibly myofibroblasts-derived foam cells led to the formation of lipid-rich core plaques, which, most often, determined lumen stenosis (Figure 4c). In contrast, no abnormal lipid deposits were visible in the C group when the hydrophobic lipid staining was performed (Figure 4a). These results are in accordance with the sera lipid profile where significant alterations were measured for both the Asi and the Au groups, with the latter case presenting the most prominent variations.

### 2.2. The Mass Spectrometric Analysis Reveals a Plethora of Alarmins with Mostly Up-Regulation Trend Caused by Hyperlipidemia

**Figure 5:**
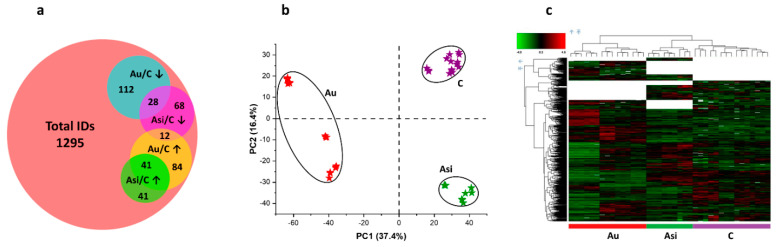
Mass spectrometry inference and relative quantification: (**a**) Venn intersection of total identifications (IDs) and the differentially abundant proteins in the Asi and Au groups, when normalized to the control values (↑—up-regulated, ↓—down-regulated); Representation of the quantitative profiles using Principal Component Analysis, (**b**) and Heat Map, (**c**) of the C (purple color), Asi (green color) and Au samples (red color). Both types of analysis demonstrate a clear distinction between the advanced hyperlipidemic proteomes (Au) as opposed to the PCSK9 siRNA-treated samples (Asi) and the control (C).

High-resolution orbitrap-based mass spectrometric analysis was performed for myocardial proteome characterization in control, hyperlipidemic, and under lipid-lowering treatment conditions. The protein inference was followed by protein FDR and Sequest score filtering, discarding contaminants, and, keeping for downstream analyses, only master-type proteins. This resulted in 1295 protein molecules passing the stringency criteria (protein FDR < 0.05 and protein Sequest score >10), (Figure 5a). We then performed label-free relative quantification using ion precursor alignment for signal intensity normalization and comparison across the three biological experimental groups. Multivariate statistics, including principal component (PCA) and hierarchical clustering analyses, were applied to visualize the global proteome differentiation. The PCA revealed clear discriminations amongst the biological quantitative features, with both having a very good spatial separation between the biological groups and homogenous technical and biological replicates (Figure 5b), and the results validated by the heat map analyses (Figure 5c).

To define the aforementioned alterations, we subsequently performed univariate statistics. In this instance, 386 proteins were thus found to be differentially abundant with a ratio >1.5 when the Asi and Au samples were compared to the C group. Most of the proteins were found to be down-regulated by the hyperlipidemic diet (112 proteins), but, interestingly, 12 proteins, which were up-regulated by hyperlipidemia, were found to be down-regulated by the combined hypolipidemic treatment. Moreover, 28 proteins were down-regulated by both conditions, while 41 presented increased spectral abundance in both situations (Figure 5a).

**Figure 6:**
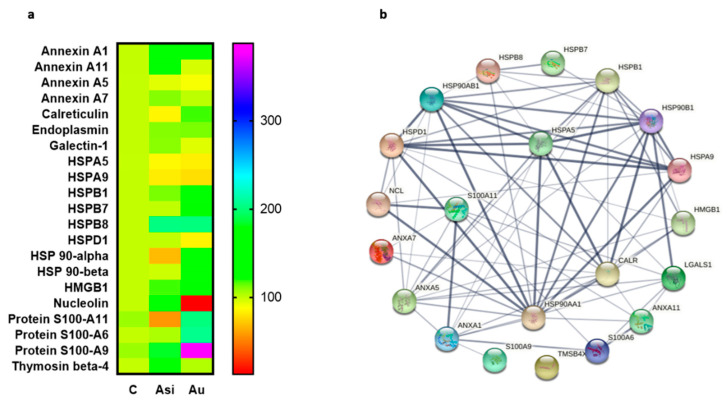
Quantitative and interaction-based profiles of identified alarmins. Heat map representation of identified alarmins (**a**) and the protein–protein interaction network and (**b**) using STRING analysis of the molecules (Heat shock proteins A5, A9, B1, B7, B8, 90AA1, 90B1, D1, nucleolin, annexin A7, thymosin β4, S100 A6, A9, and A11 proteins). The heatmap legend shows the normalized scaled-down levels of spectral abundance, while the nodes in the right network are connected using protein–protein associations or edges with confidence levels between 0.150 (low) and 0.900 (highest). All nodes are colored and signify query proteins and the first shell of interactors.

When we analyzed the damage-associated molecular patterns (alarmins) from our datasets, we found 21 alarmins that were significantly altered by either the hyperlipidemic diet or by the hypolipidemic treatment. These were: the annexin proteins A1, A5, A7, A11, calreticulin, endoplasmin, galectin-1, the heat shock proteins (HSP) A5, A9, B1, B7, B8, D1, 90-α and β, the high mobility group box 1 (HMGB1), nucleolin, the S100 proteins A6, A9, and A11, and thymosin β-4 (Figure 6a). We next applied the STRING network association to reveal the protein–protein interactions among these molecules (Figure 6b). As expected, the algorithm demonstrated a protein–protein interaction (PPI) enrichment *p*-value < 1.0 × 10^−16^ indicative of a biologically connected population of molecules. The total identified number of edges (interactions) whether text mining, experimental co-expression, or database co-occurrence was 77. HSPA5 presented the largest number of high confidence edges (6 edges with an interaction score >0.9), followed by HSP90-α (5 edges with an interaction score >0.9), and HSPA9, HSP90-β, and HSPD1 with 4 similarly high confidence edges.

**Figure 7:**
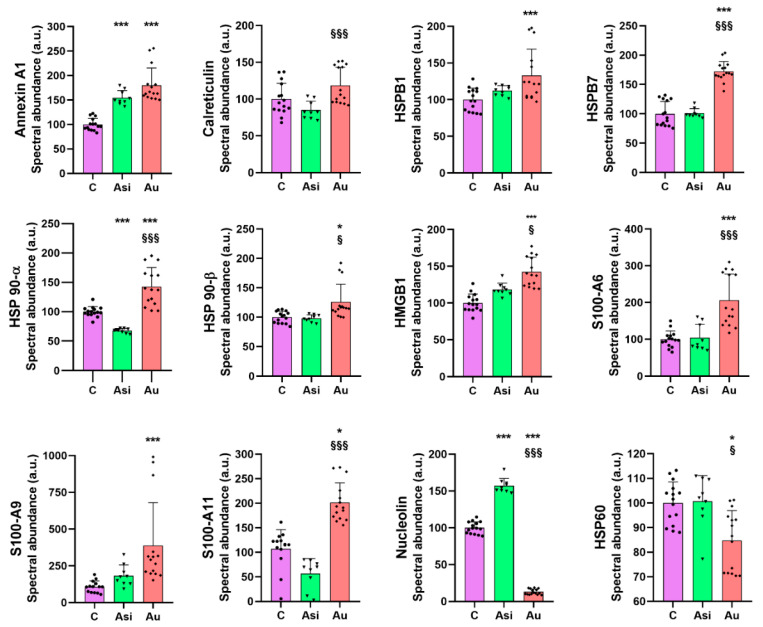
Histograms demonstrating the relative mass spectrometric normalized abundance for selected alarmins. The average ± SD of 5 biological replicates (with the exception of Asi, where *n* = 3) is plotted. The statistical significance over the C samples is represented by * (* *p* < 0.05, *** *p* < 0.001), while the one over Asi is visible with § (§ *p* < 0.05, §§§ *p* < 0.001). The scatter plot type histograms include both technological and biological values.

Amongst the analyzed molecules, we identified several regulation tendencies, caused, on the one hand, by the hyperlipidemic diet, and, on the other, by the lipid-lowering treatment. For most of the selected alarmins, the high-fat diet determined an up-regulation of the protein level translated through the mass spectrometric abundance (Figure 7). A significant up-regulation was observed when compared to the control group for HSPB1 (1.3-fold), HSP 90-α (1.4-fold), HSP 90-β (1.2-fold), and HMGB1 (1.4-fold). Significant up-regulation of >1.5-fold over the C sample was demonstrated for annexin A1 (1.8-fold), HSPB7 (1.7-fold), whereas a >2-fold alteration was found for S100 A11 (2-fold), S100 A6 (2-fold), and S100 A9 (3.6-fold). In most of these cases, we registered a non-significant regulation of the alarmin molecules in the Asi group over the C samples, with spectral abundance comparable with that of the C samples (calreticulin, HSPB1, HSPB7, HSP 90-α, HSP 90-β, HMGB1, S100-A6, S100-A9, S100-A11). In other words, a down-regulation trend of these alarmins (compared to the Au group) was observed with the administration of the combined hypolipidemic treatment. On the other hand, we noticed the opposite tendency of nucleolin and HSP60 alarmins, which were under-regulated by the high-fat diet. Of note is that the nucleolin’s spectral abundance was decreased by 7.7-fold over the C group and 12-fold over the Asi group, whereas HSP60 was down-regulated in the Au group.

### 2.3. Verification and Validation of Mass Spectrometry Results

**Figure 8:**
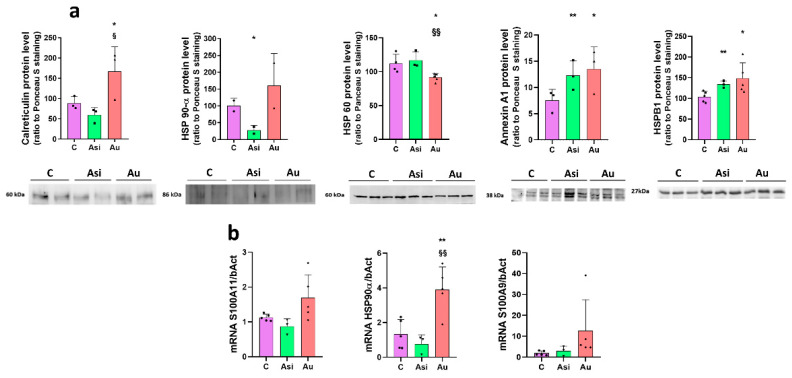
Verification and validation of mass spectrometry results through immune-based and gene expression assays. (**a**) Immunoblot experiments (lower panel) and the corresponding quantification histogram (**upper panel**) of control (C), PCSK9 siRNA-treated hyperlipidemic (Asi) and advanced hyperlipidemic (Au) samples, for calreticulin, HSP 90-α, HSP 60, Annexin A1, and HSPB1 proteins. The statistical significance over C samples is represented by * (* *p* < 0.05, ** *p* < 0.005), while the one over Asi is visible with § (§ *p* < 0.05, §§ *p* < 0.005). (**b**) Real-Time PCR relative quantitative analyses for S100A11, HSP 90-α, and S100A9 genes. The statistical significance over C samples is represented by * (** *p* < 0.005), while the one over Asi is visible with § (§§ *p* < 0.005). The scatter plot type histograms include biological values only.

Alternative, immuno-based, and gene expression assays were performed for validation and for further investigation of the mass spectrometry results. We thus performed immunoblot experiments for the validation of calreticulin, HSP 90-α, HSP60, Annexin A1, and HSPB1 alarmins. Calreticulin protein levels presented the same regulation trend as demonstrated by the mass spectrometry results. A non-significant decrease over the C group was observed for the Asi samples, while Au showed the highest protein levels, with significant up-regulation over both the Asi and C sample groups (Figure 8a). A similar alteration tendency was also uncovered for HSP 90-α, where Asi presented an even higher and significant down-regulation over C (3.6-fold), (Figure 8a). HSP 60 regulation was in line with the spectral abundance results, with significant down-regulation caused by the prolonged hyperlipidemic diet over both the control and treated hyperlipidemic samples (Figure 8a). Annexin A1 and HSPB1 Western blotting data demonstrated a significant relative difference between both hyperlipidemic groups over the control one, with non-significant alteration between the first two, a validation of the mass spectrometric results (Figure 8a).

Using Real-Time PCR experiments, we evidenced the same regulation tendencies for the S100A11 and S100A9 genes (Figure 8b). The mass spectrometric abundance alteration for HSP 90-α was validated with significant 3-fold and 5-fold over-regulation caused by hyperlipidemia over the control and treated groups, respectively (Figure 8b).

## 3. Discussion

Gene-silencing agents, such as small interfering RNA (siRNA), have been proposed as the next generation of drugs designed to antagonize PCSK9. Such an example is inclisiran, which has been implemented in various past and ongoing clinical trials with extended benefits and a proven higher efficacy over the classic monoclonal antibody-based PCSK9 inhibitors [20]. Herein, we designed and implemented rabbit-specific small interfering RNA for the inhibition of PCSK9, which, in combination with statin treatment, provides a possible synergistic effect for lowering circulating LDL-C and the subsequent stabilization of the atheromatous plaques. It is important to highlight that both hepatic and circulating levels of PCSK9 were not statistically regulated by the lipid-lowering treatment when compared to the other experimental groups. Moreover, the temporal quantification of plasma PCSK9 before, during, and after the hypolipidemic treatment, revealed the same lack of statistical relevance, albeit a decrease in the absolute amount was measured after two weeks of treatment. This, however, is expected and is in accordance with previous studies that demonstrated the PCSK9 up-regulation following statin treatment [8,21,22]. We can thus speculate a compensatory mechanism, in which, on the one hand, the statin alone up-regulates the PCSK9 biosynthesis, and, on the other, gene inhibitory effects are produced by PCSK9 siRNA therapy. The LDL receptor expression evaluation which we performed, however, was indirect evidence of PCSK9 inhibition using the siRNA approach, whereby, as expected, the higher expression in the Asi group vs. the Au group would account for a more pronounced recycling route of the receptor, the main target of action for PCSK9.

We have obtained radical and highly significant differences between the sera lipid profiles after the lipid-lowering treatment and the high-fat diet. As expected, the total cholesterol and the LDL-C sub-fraction levels were drastically reduced after the hypolipidemic treatment. Moreover, the Asi serum triglycerides level presented the highest difference vs. Au (~4-fold lower), in accordance with previous studies assessing the positive association of PCSK9 inhibition with triglycerides level [23]. To further validate our experimental setup, we performed serial sectioning of the left ventricular tissue and used the lipid-soluble dye Oil Red O for staining lipid deposits inside coronary arteries. Consistent with the circulating lipid profile, we observed stenotic arteries in the Au samples, with occluding lipid deposits inside all of the lumen vessels, with possible associated non-fatal hypoxic events, such as myocardial infarctions. In contrast, when presented with substantial deposits which determined intimal thickening, the coronary artery sections harvested from the Asi animals did not resolute into total lumen occlusion. Although not challenged to a hyperlipidemic diet for the whole three months, as was the case for the Au group, the transition to a standard diet concomitant with a strong hypolipidemic treatment for the last experimental month led to the apparent reduction of the atheromatous plaques for the Asi animals.

Even though clear and significant differences were revealed between the Asi and Au animals, a residual risk for coronary events cannot be excluded for the former experimental group. Using powerful high-resolution mass spectrometry-based proteomics, we demonstrate the protein profile differences in the myocardial tissue containing part of the left anterior-descending coronary artery from the two animal groups, after normalization to the reference one. We applied multivariate statistics to compare the proteomic profiles from the three biological groups. Both principal component analysis and hierarchical clustering heat map analysis corroborated in demonstrating distinct proteomes for the C, Au, and Asi groups. Moreover, it is worth noting that the heat map analysis also highlighted the dendrogram cluster dependency of the C and Asi groups as opposed to the Au group, demonstrating a higher degree of similarity between the treated hyperlipidemic and control groups, than the advanced hyperlipidemic one. After stringent filtering of the label-free quantitative data, we demonstrated the mass spectrometric abundance alteration of 386 proteins, which was either due to the high lipid diet alone or a high lipid diet for two-thirds of the animal experimentation period that was succeeded with a standard diet along with a combined hypolipidemic treatment.

To evaluate the association potential of alarmins with the degree of atheromatous plaque evolution, we next focused on investigating a specific subset of molecules, with “danger-” or “stress-sensing” capabilities. In this investigation, 21 alarmins were identified and quantified in our proteomic dataset, most of them from the annexin, heat shock, and S100 families of proteins, with strong interaction-based correlations as demonstrated by the network association analysis. As expected, from the total panel of alarmins, we observed a marked tendency of over-regulation caused by the extended hyperlipidemic diet, consistent with an inflammatory status in the harvested myocardial tissue [13].

Heat shock proteins are chaperone molecules, assisting in correct folding during the maturation of newly synthesized proteins. Under stressful conditions, the differential expression of some HSPs has been evidenced [24] and, specifically, their over-expression in atherosclerotic conditions [25]. In our study, we demonstrate that advanced hyperlipidemia over-regulates HSPB1, HSPB7, HSP90-α (by both mass spectrometry and immunologic assay), and HSP90-β, while HSP60’s spectral abundance was found to be down-regulated. The treatment reverses the regulation trends for HSP27 (HSPB1) and HSP60.

HMGB1 is a ubiquitous nuclear architectural factor [14], in which inflammation is secreted with direct implications for the pathogenesis of the disease. HMGB1 over-expression was demonstrated in both the nuclei and cytoplasm of macrophages and the smooth muscle cells near the intima of the coronary artery lesions [26]. A feedback loop action of HMGB1 was proposed by our group, explaining the inflammation enhancement in hyperlipidemia that might lead to the continuous long-term development of atheroma plaques [27]. Our present results are consistent with these reports, with HMGB1 being significantly up-regulated by the hyperlipidemic diet and lowered by the hypolipidemic treatment, corroborating well with previously published data that projected HMGB1 as a mortality predictor after coronary events [28,29].

Nucleolin, a multifunctional RNA-binding protein, implicated in apoptosis, cell proliferation, or microRNA processing [30], was down-regulated in the advanced plaques of murine aortas when compared with the early plaques of ApoE−/− mice [31]. Our results on myocardial tissue, where nucleolin was down-regulated 7.7-fold over the C group and 12-fold over Asi, are, therefore, in line with the previous findings and help strengthen the association of this alarmin with atherogenic pathology.

From the S100 family of calcium-binding cytosolic proteins, several members have been positively associated with an inflammatory status specific for an ischemic event, especially the S100-A8/A9 heterodimer [32]. Its expression was found to be massively increased following a myocardial infarction, with a demonstrated role in the initiation, progression, and maintenance of an inflammatory status, which rendered it a critical therapeutic player in the recovery post-MI [33]. Our findings were concurrent with these studies, identifying a significant over-regulation of S100-A6, S100-A9, and S100-A11 by the hyperlipidemic treatment. It is worth highlighting that the combined statin and PCSK9 siRNA treatment determined a decrease in protein level and a non-significant alteration over the control group.

Calreticulin, a 60 kDa ubiquitous Ca^2+^-binding protein, has been reported to regulate calcium hemostasis by inhibiting coronary thrombosis [34]. The calreticulin level demonstrated by our present proteomic results is significantly over-regulated in the advanced hyperlipidemic samples (Au group), suggesting increased inflammation. The hypolipidemic treatment applied to the Asi group significantly lowers the protein level up to a basal level.

Annexin A1, a 37 kDa pro-resolving protein, with essential membrane organization and trafficking roles [35], has been demonstrated to possess anti-inflammatory characteristics at the atheroma lesion site [36,37,38]. In our study, the cardiac lysate demonstrated significantly increased levels of annexin A1 in both hyperlipidemic conditions (Asi and Au) relative to the control, indicative of an inflammatory vascular bed.

It is important to highlight that although the results reported here are mostly consistent with previously published data, as highlighted above, there are some conflicting results that may be attributed to the molecular temporal dynamics or the site of expression with regard to the disease progression. Such is the case for HSPB1, also known as HSP27, which we found to be significantly over-regulated by the prolonged hyperlipidemic diet. Disparate findings with regard to the protein level of HSPB1 have cast a controversial shadow upon its true pathophysiological expression. While a decreased circulating level was associated with increasing plaque progression and instability [39], another study did not identify a significant association with cardiovascular events [40]. Another such example is represented by HSP60, whose over-expression in the arterial intima of atherosclerotic subjects positioned it as a proper candidate for a therapeutic vaccine [41], but our preliminary results indicated a lower level in the advanced atherosclerotic group when compared to the other two animal groups.

One limitation of the current study is the relatively small number of biological replicates for the animal group specific to the lipid-lowering treatment, which might have an impact on the robustness of the results. Nevertheless, for the majority of cases, the biological trends were consistent and in line with previously published data. Another limitation refers to the inability of our data to distinguish the alarmin origin, whether cardiomyocyte, endothelial, or immune cell-specific. Whatever the origin, however, we base our quantification results on control normalization where alarmins should only be intracellularly located without the need to trigger inflammatory signals. Therefore, any significant alarmin regulation, as indicated by our specific assays, should be the result of the two particular stimuli applied to the animals: the high-fat diet or the lipid-lowering treatment and this should account for the extracellular-specific signal, i.e., alarmin function. Moreover, validation on large cohorts of patients with individual PCSK9 inhibitor-based, statin alone or statin combined treatment is needed to further increase the significance of our results.

Collectively, our results focus on the major findings regarding the over-regulation of HSPB1, HSPB7, HSP90-α, HSP90-β, HMGB1, S100-A6, S100-A9, S100-A11, and the under-regulation of nucleolin and HSP60, concomitant with a return to a base level comparable to the control group once the hypolipidemic treatment was applied. We also found several alarmins which we could not distinguish, statistically, between the prolonged hyperlipidemic diet and the lipid-lowering treatment, as was the case for annexin A1, annexin A5, annexin A7, endoplasmin, HSPA5, HSPA9, and HSPB8. These alarmins did not respond to the hypolipidemic treatment and can be further verified and validated in larger studies in order to determine a possible residual risk even after lipid-lowering drug therapy.

## 4. Materials and Methods

### 4.1. Reagents

All following reagents and solvents were purchased from Merck Sigma-Aldrich (Darmstadt, Germany) and were of LC-MS grade, unless otherwise specified: urea, sodium deoxycholate (DOC), Trisma hydrochloride (Tris), DL-dithiothreitol (DTT), iodoacetamide (IAA), N-acetyl-L-cysteine (NAC), ethylenediaminetetraacetic acid (EDTA), water, acetone, acetonitrile, and formic acid (FA). Gold LC-MS sequencing-grade trypsin was purchased from Promega (Madison, WI, USA). Invivofectamine 3.0 reagent was purchased from Thermo Scientific (Rockford, IL, USA). Oil Red O was offered by Merck Sigma-Aldrich, while Hematoxylin QS was ordered from Vector Laboratories (Burlingame, CA, USA). Atorvastatin was bought from Terapia Ranbaxy (Cluj-Napoca, Romania). Ambion In Vivo siRNA for PCSK9 was custom designed for inhibiting the rabbit PCSK9 gene (Gene ID: 100338756) using the Ambion by Life Technologies, now a part of Thermo Scientific (Carlsbad, CA, USA) Silencer Select algorithm and Ambion In Vivo chemical modifications. The designed sense sequence (5′—>3′) was GUCGCUUUCUUAGCAAGAAtt, while the antisense sequence was UUCUUGCUAAGAAAGCGACag. The annealed reagent was thereafter HPLC purified and used in a 1.2:1 siRNA: lipid ratio as recommended by the manufacturer.

### 4.2. Experimental Animal Models

Animal experimentation was performed in the animal husbandry of “Cantacuzino” National Institute of Research and Development for Microbiology and Immunology, Baneasa branch (Bucharest, Romania). The animals were housed in a controlled temperature and humidity environment, with 12 h light cycles, and had access to water ad libitum.

Of these, 13 healthy male three-months-old New Zealand White (NZW) rabbits were randomized into three groups. A control group (C) of 5 animals consisted of NZW rabbits that received a standard chow diet for twelve weeks. The second treated group (Asi) contained 3 NZW rabbits that received a high-fat diet with 0.5% cholesterol and 5% corn oil for the first eight weeks, after which they were switched to a standard diet together with the simultaneous administration of a daily oral dose of atorvastatin (3,5 mg/kg body, 5 days per week) and one weekly IV dose of PCSK9 siRNA-based inhibitor (0.15 mg/kg body) for the following four weeks. The last group of 5 NZW rabbits was the vulnerable atherosclerotic group (Au), which received a high-fat diet with 0.5% cholesterol and 5% corn oil for the entire twelve weeks of animal experimentation. In the end, the heart and both auricular and ventricular blood were collected and stored appropriately for further analysis.

### 4.3. Sera Biochemical Determinations

Sera determinations of total cholesterol (CHOD-PAP method), LDL-cholesterol (enzymatic selective protection), triglycerides (GPO-PAP method), and glucose (GOD-PAP method) were colorimetrically performed using specific assay kits, provided by Dialab (Vienna, Austria). The associated absorbance measurements were performed with the Pherastar FS microplate multimodal reader (BMG Labtech, Ortenberg, Germany).

### 4.4. Rabbit PCSK9 Enzyme-Linked Immunosorbent Assay

PCSK9 absolute level determinations were performed using a specific kit purchased from Fine Test (Wuhan, China), which was based on a sandwich technology where anti-PCSK9 antibody were pre-coated onto 96-well plates and biotin-conjugated anti-PCSK9 used as detection antibodies. The working procedure was performed according to the manufacturer’s indications. Briefly, the plate was washed twice with a specific buffer before adding 100 µL of either sera or diluted hepatic (up to 5 µg/µL total protein) samples (3 biological replicates, each with technological duplicates) and the corresponding standards were incubated for 90 min at 37 °C. The plate was then aspirated and washed twice, followed by the addition of 100 µL biotin-labeled antibody working solution to each well and incubated for 60 min at 37 °C. Thrice plate aspiration and washing were proceeded by incubation for 30 min at 37 °C with 100 µL of HRP-Streptavidin Conjugate working solution. Another 5 steps of aspiration and plate washing were followed by TMB substrate addition (90 µL) and incubation for 30 min at 37 °C. Afterward, Stop solution was added (50 µL) and 450 nm absorbance measurements were performed using the Pherastar FS system.

### 4.5. Histological Assessment of Atherosclerotic Plaques

Cardiac inter-ventricular fragments containing the left-descending coronary artery were collected from each animal group and suitably processed for microscopy histological investigations. The tissue fragments were immersed in an OCT embedding medium for 15 min at room temperature and then flash-frozen in liquid nitrogen. Cryosections were collected using the Leica CM 1850 cryotome (Wetzlar, Germany) and stained with Oil Red O and hematoxylin-eosin, as previously described [42], mounted in 90% glycerol in water, and examined using the Zeiss AXIOVert A1 microscope (Zeiss LD-Plan-Neofluar 20×/0.4 Ph2 Korr objective lens, Zeiss, Oberkochen, Germany). The histological images were captured with the Zeiss AXIOcam MRc5 Camera using ZEN imaging software (v. 2012, Blue Edition).

### 4.6. Liquid Chromatography—Mass Spectrometric Analysis

Cardiac cryosections of 30 mg of inter-ventricular myocardial tissue containing part of the left-descending coronary artery was homogenized in 0.3 mL lysis buffer containing 8M urea, 1% DOC, and 100 mM Tris-HCl (pH 7.5) and Protease Inhibitor Cocktail I (Merck, Darmstadt, Germany), on ice, using a rotor-stator mechanical homogenizer (Polytron PT 1300D, Kinematica, Malters, Switzerland). Following powerful centrifugation (10,000× *g*, 10 min, and 4 °C), the protein supernatant was used for protein level determination (Pierce BCA Protein Assay, Thermo Scientific). Thereafter, 50 µg of protein from each sample were purified through acetone precipitation (1:4 ratio of protein sample to ice-cold acetone, 2 h, −28 °C), separated after a 20 min, 20,000× *g* centrifugation at 4 °C, and, after the supernatant was discarded, the protein pellet was resuspended in a reducing buffer containing 8 M urea, 0.1 M Tris-HCl, 0.1 mM EDTA, and 20 mM DTT. The cysteine residues were alkylated using IAA (80 mM), followed by NAC (80 mM) quenching. Over-night proteolysis was performed using sequencing-grade-modified trypsin in a 1:20 enzyme to substrate ratio at 37 °C under basic conditions (pH 8.5). For salt removal, we purified the peptides through solid phase extraction using C18 Sep-Pak columns (Waters Corporation, Milford, DE, USA). The eluate was evaporated using the Concentrator Plus system from Eppendorf (Hamburg, Germany) and the dried peptide mixtures were resuspended in a solution containing 5% acetonitrile, 0.1% formic acid in LC-MS grade water. We used the Pierce Quantitative Colorimetric Peptide Assay (Thermo Scientific) to determine peptide quantity and inject equal amounts from each sample (1 µg/replicate).

Liquid nano-chromatography experiments were realized using the Easy nLC II system (Thermo Scientific), while mass spectrometry was performed using the LTQ Orbitrap Velos Pro ETD system (Thermo Scientific). The peptide samples were injected in triplicate and separated using a 3–25% solvent B (0.1% formic acid in acetonitrile) over A (0.1% formic acid in water) using an Easy 10 cm × 75 μm·d., C18, 3 μm, 120 Å analytical column (Thermo Scientific) at a flow rate of 300 nL/min. The MS was operated in a top 15 data-dependent configuration at 60 k resolving power for a full scan across the 350–1700 m/z domain with a collision-induced dissociation (CID) fragmentation mode for MS2. Liquid junction was preferred as the interface to the ion transfer tube, using a 12 cm length, 360 μm outer diameter, 20 μm inner diameter, and 10 μm tip inner diameter uncoated PicoTip emitter (New Objective, Woburn, MA, USA). The set voltage was 1.6 kV and an ion transfer tube temperature of 275 °C was selected.

### 4.7. Bioinformatic Analysis

Proteome Discoverer 2.4 (Thermo Scientific) was used for protein inference and relative quantification. Sequest HT was preferred as a search engine in the Oryctolagus cuniculus Uniprot database. Methionine oxidation was set as dynamic modification and cysteine carbamidomethylation as a static one, allowing two missed cleavages for in silico proteolysis. An in-house contaminant database was used to recognize and filter out common protein contaminants. Label-free relative quantification was performed using the precursor ion quantifier node and was based on the intensity of the unique peptide precursors from 90% of the replicate features. Normalization was performed on the total peptide amount, while the scaling parameter was set on the control average. Protein abundances were calculated as the average of the most abundant distinct peptide groups, while the protein ratio was directly calculated from the grouped protein abundances. The statistical significance of the quantification ratio comparison was calculated using the ANOVA hypothesis test and corrected using the Benjamini–Hochberg FDR-based algorithm. STRING freeware (v.11.0) was used for creating the interaction-based networks and accession to KEGG (Kyoto Encyclopedia of Genes and Genomes) and Gene Ontology databases.

### 4.8. Western Blot Experiments

Equal amounts of protein samples were separated using the SDS-PAGE technique and afterward transferred onto nitrocellulose membranes. Ponceau S staining was used for electro-transfer uniformity and normalization purposes. Thereafter, the membranes were washed and blocked with 2% BSA in TRIS-buffered saline containing 0.05% Tween 20, pH 7.6, and exposed for 2 h to the primary calreticulin (Thermo Scientific, catalog # PA3-900), HSP 90-α (Abcam, Cambridge, UK, catalog # ab13492), HSP 60 (Thermo Scientific, catalog # MA3-013), HSPB1 (Abcam, catalog # ab79868), and Annexin A1 (Thermo Scientific, catalog # PA5-22266) antibodies in TBS with 1% BSA followed by the appropriate IgG coupled with horse radish peroxidase (IgG–HRP) secondary antibodies for 1 h (Abcam, catalog # ab6721, and Sigma-Aldrich, catalog # A2304). The subsequent chemiluminescence reaction was revealed using the ECL Western Blotting Substrate kit (Thermo Scientific) and images were taken with the Image Quant LAS 4000 camera system (GE Healthcare, Uppsala, Sweden). Thereafter, Digital densitometry analysis was performed using the ImageJ analysis freeware. All original, unaltered, and unprocessed membrane and Ponceau S-colored membrane images can be found in Figures S1–S6 as Supplementary Material.

### 4.9. Gene Expression Analyses

Total RNA was extracted from 90 mg of left heart ventricle or hepatic tissue using the RNeasy Mini Kit (QIAGEN, Hilden, Germany). The quality of isolated nucleic acid was assessed using an Agilent 2100 Bioanalyzer (Agilent Technologies, Santa Clara, CA, USA) and quantification was performed by Nano Drop ND 1000 absorbance measurements (Thermo Scientific). Thereafter, 1 μg of total RNA was used to generate cDNA with a Transcriptor First Strand cDNA Synthesis Kit (Roche, Mannheim, Germany). We used the LightCycler 480 SYBR Green I Master mix to perform qPCR in the Light Cycler System (Roche). Triplicate reactions were performed, while product specificity was verified by melting curve analysis. Amplification of the housekeeping gene β-Actin was used for normalization. The primer sequences selected are as follows: protein S100A11 forward, 5′-CCGTGTTCCAGAAGTACGCT-3′ and reverse, 5′-CTTCATCATGCGGTCGAGGA-3′, protein heat shock protein 90α (HSP90α) forward, 5′-GAACCAGCTTGACGGAGGAA-3′ and reverse, 5′-CCCACAAACCTCGGTGACTT-3′, protein S100A9 forward, 5′-ATCTGTGGGCTCCTCTGCTTT-3′ and reverse, 5′-TCCCTCGCCTCCTTCTTGAG-3′ and protein β-actin forward, 5′-GTGCTTCTAGGCGGACTGTT-3′ and reverse 5′-CGGCCACATTGCAGAACTTT-3′, protein LDL receptor forward, 5′-GGTGAACTGGTGCGAGAAGC-3′ and reverse, 5′-GAACTTGGGCGAGTGGCTAT-3′. Light Cycler 480 Software using the Ε (Efficiency) method was used for gene expression relative quantification.

### 4.10. Other Statistical Analyses

The results were expressed as a mean ± standard deviation (SD). The data were analyzed by the student’s unpaired *t*-test algorithm or one-way ANOVA within GraphPad Prism 8.0.1 software (GraphPad Software, CA, USA), wherein the significance threshold (*p*-value) was set <0.05.

## 5. Conclusions

In conclusion, we report that:

1. This is the first proteomic study focusing on a panel of cardiac alarmins and their alteration in hyperlipidemia;
2. Specific alarmins from the annexin and heat shock families could not be stabilized by the lipid-lowering therapy and may be included in further functional studies to determine the atherosclerotic residual risk.

## Data Availability

The Mass spectrometry data were deposited in the PRIDE [43] repository via ProteomeXchange [44] with the dataset identifier PXD035692.

## Supplementary Materials

The supporting information can be downloaded at: https://www.mdpi.com/article/10.3390/ijms231911174/s1.
