# Supplementary material for: Cardiac Alarmins as Residual Risk Markers of Atherosclerosis under Hypolipidemic Therapy
Source: Int J Mol Sci. 2022 Sep 22;23(19):11174. doi: 10.3390/ijms231911174 (PMC9569654; doi:10.3390/ijms231911174)
Supplement: Supplementary file 1 [file ijms-23-11174-s001.zip › ijms-1877663-supplementary.pdf]

Original Western blot images used for protein relative quantification

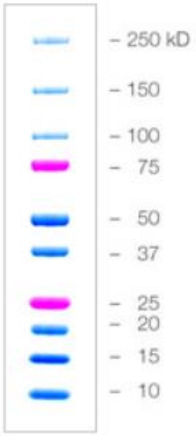

Figure S1: Bio-Rad Dual Color Protein Ladder (catalog #1610374) that was used for referencing the molecular weight during electrophoresis and after membrane transfer

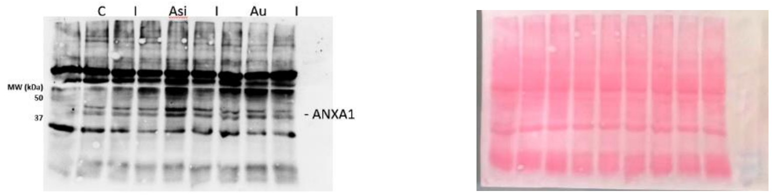

Figure S2: Original (uncropped and unaltered) membrane image and associated Ponceau S colored membrane, used for the relative quantification through Western blot technique of Annexin A1 antigen, in the rabbit cardiac tissue lysate of control (C), treated hyperlipidemic (Asi) and hyperlipidemic samples (Au).

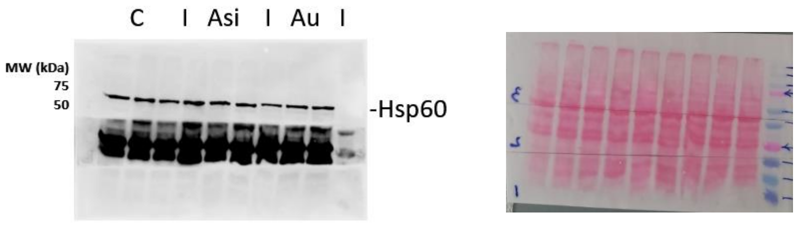

Figure S3: Original (uncropped and unaltered) membrane image and associated Ponceau S colored membrane used for the relative quantification through Western blot technique of Hsp60 antigen, in the rabbit cardiac tissue lysate of control (C), treated hyperlipidemic (Asi) and hyperlipidemic samples (Au).

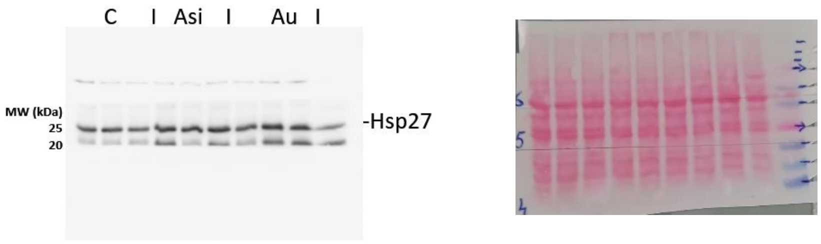

Figure S4: Original (uncropped and unaltered) membrane image and associated Ponceau S colored membrane used for the relative quantification through Western blot technique of Hsp27 antigen, in the rabbit cardiac tissue lysate of control (C), treated hyperlipidemic (Asi) and hyperlipidemic samples (Au).

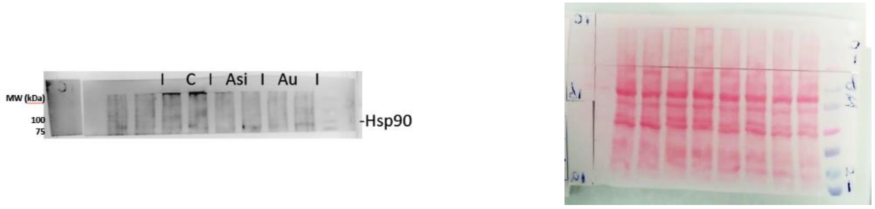

Figure S5: Original (uncropped and unaltered) membrane image and associated Ponceau S colored membrane used for the relative quantification through Western blot technique of Hsp90 antigen, in the rabbit cardiac tissue lysate of control (C), treated hyperlipidemic (Asi) and hyperlipidemic samples (Au).

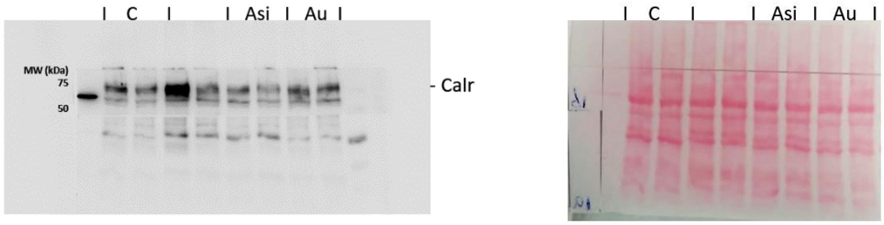

Figure S6: Original (uncropped and unaltered) membrane image and associated Ponceau S colored membrane used for the relative quantification through Western blot technique of calreticulin antigen, in the rabbit cardiac tissue lysate of control (C), treated hyperlipidemic (Asi) and hyperlipidemic samples (Au).
